# Genetic and Phenotypic Parameters for Pelt Quality and Body Length and Weight Traits in American Mink

**DOI:** 10.3390/ani12223184

**Published:** 2022-11-17

**Authors:** Shafagh Valipour, Karim Karimi, David Barrett, Duy Ngoc Do, Guoyu Hu, Mehdi Sargolzaei, Zhiquan Wang, Younes Miar

**Affiliations:** 1 Department of Animal Science and Aquaculture, Dalhousie University, Truro, NS B2N 5E3, Canada; 2 Department of Pathobiology, University of Guelph, Guelph, ON N1G 2W1, Canada; 3 Select Sires Inc., Plain City, OH 43064, USA; 4 Livestock Gentec, Department of Agricultural, Food and Nutritional Science, University of Alberta, Edmonton, AL T6G 2H1, Canada

**Keywords:** American mink, pelt quality, body size, genetic parameters, heritability

## Abstract

**Simple Summary:**

The present study estimated the heritability, phenotypic and genetic correlations for different pelt quality measures, body weight and length traits in mink. Body weight and length measured on live animals in November of the first year of life were reliable indicators of dried pelt size, without negative impacts on pelt quality traits. In addition, body length in November and harvest had moderate positive genetic correlations with dried pelt quality, which made this trait an appealing trait to select for increased pelt size. The estimated genetic parameters for these traits can be used to improve fur characteristics in Canadian mink populations.

**Abstract:**

Understanding the genetics of fur characteristics and skin size is important for developing effective breeding programs in the mink industry. Therefore, the objectives of this study were to estimate the genetic and phenotypic parameters for pelt quality traits including live grading overall quality (LQU), live grading nap size (LNAP), dried pelt size (DPS), dried pelt nap size (DNAP) and overall quality of dried pelt (DQU), and body length and weight traits, including November body weight (Nov_BW), November body length (Nov_BL), harvest weight (HW) and harvest length (HL) in American mink. Dried pelt quality traits on 1195 mink and pelt quality traits on live animals on 1680 were collected from mink raised at two farms, in Nova Scotia and Ontario. A series of univariate analyses were implemented in ASReml 4.1 software to identify the significance (*p* < 0.05) of random effects (maternal genetic effects, and common litter effects) and fixed effects (farm, sex, color type, year, and age) for each trait. Subsequently, bivariate models were used to estimate the genetic and phenotypic parameters using ASReml 4.1. Heritability (±SE) estimates were 0.41 ± 0.06 for DPS, 0.23 ± 0.10 for DNAP, 0.12 ± 0.04 for DQU, 0.28 ± 0.06 for LQU, 0.44 ± 0.07 for LNAP, 0.29 ± 0.10 for Nov_BW, 0.28 ± 0.09 for Nov_BL, 0.41 ± 0.07 for HW and 0.31 ± 0.06 for HL. DPS had high positive genetic correlations (±SE) with Nov_BW (0.89 ± 0.10), Nov_BL (0.81 ± 0.07), HW (0.85 ± 0.05) and HL (0.85 ± 0.06). These results suggested that body weight and length measured on live animals in November of the first year were reliable indicators of dried pelt size. DQU had favorable genetic correlations with Nov_BL (0.55 ± 0.24) and HL (0.46 ± 0.20), and nonsignificant genetic correlations with DNAP (0.13 ± 0.25), Nov_BW (0.25 ± 0.25) and HW (0.06 ± 0.20), which made body length traits an appealing trait for selection for increased pelt size. High positive genetic correlation (±SE) was observed between LNAP and DNAP (0.82 ± 0.22), which revealed that nap size measurement on live animals is a reliable indicator trait for dried pelt nap size. However, nonsignificant (*p* > 0.05) low genetic correlation (±SE) was obtained between LQU and DQU (0.08 ± 0.45), showing that indirect selection based on live grading might not lead to the satisfactory improvement of dried pelt overall quality. The estimated genetic parameters for live grading, dried pelt quality, and body weight and body length traits may be incorporated into breeding programs to improve fur characteristics in Canadian mink populations.

## 1. Introduction 

American mink (*Neogale vison*) is the most favored fur-bearing animal being raised under an intensive production system. Mink farming was initiated in Canada in 1866 [1], and since then, due to its importance for fur industries, mink farming was extensively practiced in North America, Europe and Asia [2,3]. The quality of fur and pelt size are the main factors that determine the final price of pelt and subsequently the profitability of mink producers [4]. Therefore, there is an increasing interest in breeding mink with more desirable fur characteristics. Pelts with larger size, higher density of hair and healthy appearance of guard hair gain the highest economic value [5,6]. 

Pelt quality is a composite trait that includes pelt nap size, underfur density, silky appearance of fur, guard hair thickness and purity of color of underfur [7]. In the current commercial evaluation system of mink pelt, pelt nap size (NAP) and overall quality of fur are the most used objective traits for genetic improvement of pelt quality. The proportion of guard hair (long stiff hairs) that protrudes out of the underfur (thin-short hairs) is referred to as the nap causing the wavy and shiny appearance of fur, and clothing industries and consumers demand short nap fur [7,8,9]. 

The evaluation of fur characteristics can be performed on both live mink (grading traits) and dried skins (pelt traits). Live grading is subjective and performed by certified technicians on the farm, while evaluation of fur characteristics is performed on dried pelt using sorting machines by certified fur grading specialists in auction houses. Although the price of pelts is highly determined based on pelt quality traits, these traits are not available until post-harvest. Alternatively, live grading and body weight and length measurements can be used as potential indicators of dried pelt characteristics [10,11]. Therefore, evaluating animals for both live grading and dried pelt traits are important for selection of mink with better quality of fur. Moreover, assessing the correlations between live grading and dried pelt quality traits should be considered for designing a successful breeding program. 

Canada is a major producer and exporter of mink pelt in the world with a production record of 1.76 million pelts in 2018 [12]; however, a comprehensive genetic breeding program has not been implemented by the Canadian mink industry. Farmers select animals phenotypically with higher litter size and better fur characteristics as parents of the next generation. However, phenotypic selection is not an effective method to improve performance of animals because a large portion of phenotypic variation of traits are explained by environmental and non-additive genetic effects that are not transmissible to the next generation [13]. On the other hand, genetic selection for pelt quality traits requires estimating the heritabilities as well as the phenotypic and genetic correlations among these traits. Several studies have estimated the genetic parameters and heritabilities for live grading traits in American mink populations [14,15,16,17,18,19,20]. However, heritabilities for dried pelt quality traits and their genetic correlations with live grading traits and body weight and length traits have rarely been reported [6,11]. To our knowledge, no study has estimated the genetic and phenotypic parameters for pelt quality traits on dried pelt and their genetic and phenotypic correlations with live grading traits in Canadian mink populations. Therefore, the objectives of this study were as follows: (1) to estimate the heritabilities for three dried pelt quality traits (dried pelt size, dried pelt nap size and overall quality of dried pelt), (2) to estimate genetic and phenotypic correlations between dried pelt quality traits and live grading traits (live grading nap size and live grading overall quality) and (3) to estimate genetic and phenotypic correlations between pelt quality traits and four body size measurements, including November body weight, November body length, harvest weight and harvest length in mink.

## 2. Material and Methods

The present study was approved by the Dalhousie University Animal Care and Use Committee (certification#: 2018-009, and 2019-012). Moreover, the mink used in this research work were cared for following the Code of Practice for the Care and Handling of Farmed Mink guidelines (https://www.nfacc.ca/pdfs/codes/mink_code_of_practice.pdf accessed on 25 March 2018).

### 2.1. Animals and Management

Phenotypic records used in this study were collected from animals at the Canadian Centre for Fur Animal Research (CCFAR) at Dalhousie University, Faculty of Agriculture (Truro, NS, Canada) and Millbank Fur farm (Rockwood, ON, Canada). The animals consisted of five color types, including dark, demi, mahogany, pastel and stardust. All mink were raised under standard farming conditions and had ad libitum access to food and water. The diets were formulated based on the nutrient requirements of animals in each production period. No formal breeding program was used in CCFAR or Millbank Fur Farm. Before each breeding season, weak and infertile animals were culled from the herd in December, and those with an adequate score for live grading, disease history, and reproductive records were kept for breeding. The pedigree file included 25,688 animals (1155 founder and 24,533 nonfounder individuals) and was traced through 16 generations. 

### 2.2. Evaluation of Fur Characteristics on Live Animals and Dried Pelts

Pelt quality traits were assessed on live animals (*n* = 1608) for mink in CCFAR at the end of November in 2018, 2019 and 2021 when the coat was in prime condition. Live quality grading of pelage was performed on these mink using the North American Fur Auctions (NAFA) live animal grading procedure by their certified technician. Live grading traits included the overall quality of fur (LQU) and nap size (LNAP). LQU was scored into three categories from 1 (poor) to 3 (best). LNAP was measured as the length of guard hair protruding from the underfur and scored into five categories from 1 (long) to 5 (short). 

Mink were euthanized in December 2018 and 2019 (*n* = 1195); for mink in Millbank, pelting was carried out in a pelting facility located in Millbank Fur Farm (Rockwood, ON, Canada) and mink from CCFAR were sent to custom pelting facilities (Arcadia, NS, Canada). The dried raw pelts were shipped to the North American Fur Auctions-NAFA (Toronto, ON, Canada) and Saga Furs (Vantaa, Finland) auction houses for fur quality evaluation and sale. Dried pelt quality traits included dried pelt size (DPS), dried pelt nap size (DNAP) and overall quality of dried pelt (DQU). Evaluation of DPS and DNAP was performed using sorting machines on dried skins. All skins were stretched with differential weights to adjust skin lengths into categories. DPS was measured from the tip of the nose to the base of the tail. DPS was classified into nine categories of 47.1–53 cm (category 1), 53.1–59 cm (category 2), 59.1–65 cm (category 3), 65.1–71 cm (category 4), 71.1–77 cm (category 5), 77.1–83 cm (category 6), 83.1–89 cm (category 7), 89.1–95 cm (category 8) and 95.1–101 cm (category 9). DNAP was scored into eight categories: 8 (extra short nap), 7 (short nap), 6 (short-medium open), 5 (short-medium nap), 4 (short-medium to medium nap), 3 (medium nap), 2 (medium nap to medium-long nap) and 1 (medium-long nap). DQU was assessed by professional fur grading experts in auction houses and classified into four categories: 

(1) Bronze: weakest grade assigned to the pelts, no damages, but weakest in terms of underfur, guard hair or general appearance. These pelts were very flat, coarse, weak and loose; 

(2) Silver: these pelts were complete and prime and had silky appearances. However, they had weaker underfur and poorer or uneven coverage or coarser guard hair; 

(3) Silver to golden: higher quality than silver pelts but not as good golden quality furs; 

(4) Golden: these pelts had very high-quality with good and even guard hair coverage and dense underfur. These pelts were fully prime and had smooth appearances and silky textures. 

### 2.3. Body Weight and Length Measurement

November body weight (Nov_BW) and November body length (Nov_BL) were measured on live animals at approximately seven months of age (*n* = 1734) in mid-November 2018 and 2019. Nov_BL was measured from the snout to the tail base of each mink. Following euthanasia in December 2018 and 2019, harvest body weight (HW) and harvest body length (HL) were collected from 2162 mink. HW was obtained by measuring the weight of a whole body of animals, and HL was the length of body measured from the snout to the base of tail on a whole body of mink. 

### 2.4. Statistical Analyses

The significant influence (*p* < 0.05) of nongenetic factors, including fixed effects of farm (CCFAR and Millbank Fur farm), year (2018, 2019, and 2021), sex (female and male), color type (dark, demi, mahogany, pastel and stardust) and age (1 and 2 years), and random effects of additive genetics, maternal genetics and common litter were tested for studied traits using univariate models implemented in ASReml 4.1 [21] Only significant (*p* < 0.05) effects were included in subsequent mixed model analyses. The following general univariate model was used:

y=Xb+Za+Gm+Wc+e,   

where ***y*** is the vector of phenotypic observations; ***b*** is the vector of fixed effects; ***a*** is the vector of random additive genetic effects; ***m*** is the vector of random maternal genetic effects; ***c*** is the vector of common litter effects and ***e*** is the vector of residual effects; and ***X***, ***Z***, ***G*** and ***W*** were the incidence matrices relating the phenotypic observations to fixed, random additive genetic, maternal genetic and common litter effects, respectively. It was assumed that random effects were independent and normally distributed:

$$a\sim N\left(0,\;A\sigma_{a}^{2}\right),\;m\sim N\left(0,\;A\sigma_{m}^{2}\right),\;c\sim N\left(0,I\sigma_{c}^{2}\right),\;\mathrm{and}\;e\sim N\left(0,\;I\sigma_{e}^{2}\right),$$

where ***A*** is the numerator relationship matrix; ***I*** is an identity matrix; $\sigma_{a}^{2}$, $\sigma_{m}^{2}$, $\sigma_{c}^{2}$ and $\sigma_{e}^{2}$ are the variances of random additive genetic, maternal genetic, common litter and residual effects. Bivariate models were used to estimate the genetic and phenotypic correlations between traits using ASReml 4.1 software [21].

The likelihood ratio test was used to determine the significance of different random terms in the mixed model analyses using ASReml 4.1 [21] by comparing the difference in logarithmic likelihoods between full and reduced models using the following statistics:

$$-2\left(\log L_{\mathrm{reduced}\;\mathrm{model}}-\log L_{\mathrm{full}\;\mathrm{model}}\right)\sim \chi_{\mathrm{df}\left(\mathrm{full}\;\mathrm{model}\right)-\mathrm{df}\left(\mathrm{reduced}\;\mathrm{model}\right)}^{2}$$

Relevant significant fixed and random effects were included in bivariate analyses for each trait (Table 1). Generally, the following bivariate model was used to analyze the traits:

$$\left[\begin{array}{c} y_{1}\\ y_{2} \end{array}\right]=\left[\begin{array}{cc} X_{1} & 0\\ 0 & X_{2} \end{array}\right]\left[\begin{array}{c} b_{1}\\ b_{2} \end{array}\right]+\left[\begin{array}{cc} Z_{1} & 0\\ 0 & Z_{2} \end{array}\right]\left[\begin{array}{c} a_{1}\\ a_{2} \end{array}\right]+\left[\begin{array}{cc} G_{1} & 0\\ 0 & G_{2} \end{array}\right]\left[\begin{array}{c} m_{1}\\ m_{2} \end{array}\right]+\left[\begin{array}{cc} W_{1} & 0\\ 0 & W_{2} \end{array}\right]\left[\begin{array}{c} c_{1}\\ c_{2} \end{array}\right]+\left[\begin{array}{c} e_{1}\\ e_{2} \end{array}\right],$$

where $y_{1}$ and $y_{2}$ are the vectors of phenotypic observations for trait 1 and trait 2, respectively; $b_{1}$, $b_{2}$, $a_{1}$, $a_{2}$, $m_{1}$, $m_{2}$, $c_{1}$, $c_{2}$, $e_{1}$, and $e_{2}$ are the vectors of fixed, additive genetic, maternal genetic, common litter and residual effects for trait 1 and trait 2, respectively, and $X_{1}$,$\;X_{2}$,$\;Z_{1}$,$\;Z_{2}$,$\;G_{1}$**,**$\;G_{2}$,$\;W_{1}$,$\;W_{2}$, are the incidence matrices relating phenotypic observations to fixed, random additive genetic, maternal genetic and common litter effects for traits 1 and 2, respectively. It was assumed that random effects were normally distributed:

$$\;\;\left[\begin{array}{c} a_{1}\\ a_{2} \end{array}\right]\sim N\left(0,\;A⊗\;\left[\begin{array}{cc} \sigma_{a_{1}}^{2} & \sigma_{a_{1}a_{2}}\\ \sigma_{a_{1}a_{2}} & \sigma_{a_{2}}^{2} \end{array}\right]\right),$$

$$\left[\begin{array}{c} m_{1}\\ m_{2} \end{array}\right]\sim N\left(0,\;A⊗\;\left[\begin{array}{cc} \sigma_{m_{1}}^{2} & \sigma_{m_{1}m_{2}}\\ \sigma_{m_{1}m_{2}} & \sigma_{m_{2}}^{2} \end{array}\right]\right),\;$$

$$\left[\begin{array}{c} c_{1}\\ c_{2} \end{array}\right]\sim N\left(0,\;I⊗\;\left[\begin{array}{cc} \sigma_{c_{1}}^{2} & \sigma_{c_{1}c_{2}}\\ \sigma_{c_{1}c_{2}} & \sigma_{c_{2}}^{2} \end{array}\right]\right),\;\mathrm{and}$$

$$\left[\begin{array}{c} e_{1}\\ e_{2} \end{array}\right]\sim N\left(0,\;I⊗\;\left[\begin{array}{cc} \sigma_{e_{1}}^{2} & \sigma_{e_{1}e_{2}}\\ \sigma_{e_{1}e_{2}} & \sigma_{e_{2}}^{2} \end{array}\right]\right),\;$$

where ***A*** is the numerator relationship matrix; ***I*** is an identity matrix; $\sigma_{a_{1}}^{2}$, $\sigma_{a_{2}}^{2}$, $\sigma_{m_{1}}^{2}$, $\sigma_{m_{2}}^{2}$, $\sigma_{c_{1}}^{2}$, $\sigma_{c_{2}}^{2}$, $\sigma_{e_{1}}^{2}$ and $\sigma_{e_{2}}^{2}$ are the variances of random additive genetic, maternal genetic, common litter and residual effects for traits 1 and 2, respectively; $\sigma_{a_{1}a_{2}}$, $\sigma_{m_{1}m_{2}}$, $\sigma_{c_{1}c_{2}}$ and $\sigma_{e_{1}e_{2}}$ are the covariances of random additive genetic, maternal genetic, common litter and residual effects between traits 1 and 2, respectively. Phenotypic variance was calculated as $\sigma_{P}^{2}=\sigma_{a}^{2}+\sigma_{c}^{2}+\sigma_{e}^{2}$ for HL, $\sigma_{P}^{2}=\sigma_{a}^{2}+\sigma_{m}^{2}+\sigma_{e}^{2}$ for DNAP, LNAP, Nov_BW and Nov_BL and $\sigma_{P}^{2}=\sigma_{a}^{2}+\sigma_{e}^{2}$ for other traits. Heritability of direct additive genetic effects ($h_{a}^{2}$), heritability of maternal genetic effects ($h_{m}^{2}$) and proportion of common litter variance ($c^{2}$) were defined as follows:

$$h_{a}^{2}=\frac{\sigma_{a}^{2}}{\sigma_{P}^{2}},$$

$$h_{m}^{2}=\frac{\sigma_{m}^{2}}{\sigma_{P}^{2}},\;\mathrm{and}$$

$$c^{2}=\frac{\sigma_{c}^{2}}{\sigma_{P}^{2}}.$$

Phenotypic and genetic correlations among traits were calculated using (co)variance components estimated by bivariate models.

## 3. Results and Discussion

### 3.1. Descriptive Statistics

Genetic and phenotypic parameters for dried pelt, live grading and body weight and length traits were estimated using animal models. The number of records, mean, range, standard deviation and coefficient of variation are presented in Table 2.

The LQU, HW and Nov_BL had the highest coefficient of variation (CV) among the studied traits (35.14%, 33.48% and 31.19%, respectively). The presence of these variations indicated that there might be potential to improve them through genetic/genomic selection. In the present study, the estimated CVs for DQU, LNAP and LQU were 22.87%, 30.94% and 35.14%, respectively (Table 2). Thirstrup et al. (2017) reported a higher CV of 34.53% for DQU and lower CVs of 27.43% and 25.73% for LNAP and LQU, respectively. The population used in our study consisted of five color types, while the mink used by Thirstrup et al. [6] were all standard brown color mink; this difference in population structure might be the reason for higher genetic variation in our study relative to that in their study.

### 3.2. Fixed and Random Effects

In order to determine the influence of nongenetic factors on phenotypic variation of the traits, the significance of fixed effects and nongenetic environmental effects were examined using univariate animal models. The fixed effects of farm and year were significant (*p* < 0.05) for all pelt and body weight and length traits (Table 1). The fixed effect of sex was significant (*p* < 0.05) for DPS, Nov_BW, Nov_BL, HW and HL. This is in agreement with a previous investigation that reported a difference in mature body weight and body length between female and male mink [22]. The effect of color type was significant for DNAP, LNAP, LQU, Nov_BL and HL (Table 1). The difference in body weight of mink at maturity among different color types has been previously reported in a study of the growth pattern of mink [23].

Estimated variance components for each trait obtained from univariate models are presented in Table 3. The maternal genetic effects (±SE) were significant (*p* < 0.05) for DNAP, LNAP, Nov_BW and Nov_BL (Table 1) and explained 0.15 ± 0.06, 0.07 ± 0.03, 0.19 ± 0.04 and 0.15 ± 0.04 of the phenotypic variation for these traits, respectively. These results revealed that maternal genetic effects might be an important determinant of phenotypic variation for nap size, body weight and body length. Selection of animals for maternal ability by culling out dams with weak maternal ability on these traits will then have positive effects on both body weight and length and pelt quality. There were no previous reports on the significance of random maternal genetic effects on pelt traits and body weight and body length traits in mink for comparison. However, in Alpine Merino sheep, maternal genetic effects were significant (*p* < 0.05) for yearling staple length (0.03) and yearling body weight (0.18) [24].

The random common litter effect was only significant (*p* < 0.05) for HL (0.09 ± 0.03). The proportion of common litter effects was not tested for live grading and pelt traits since there were no common dams in different years of data collection for these traits. However, previous studies reported a minor contribution of common litter effects on live grading and pelt traits in mink, ranging from 0.01 to 0.12 [6,15].

### 3.3. Heritability Estimations

Heritability estimations for all traits obtained from bivariate models are presented in Table 4 (diagonal elements). The average value of bivariate estimations of heritabilities was similar to those obtained by univariate analyses. Minor differences between these estimates can be due to the differences in the number of records available for some traits.

The estimated heritabilities (±SE) were 0.41 ± 0.06 for DPS, 0.29 ± 0.10 for Nov_BW, 0.28 ± 0.09 for Nov_BL, 0.41 ± 0.07 for HW and 0.31 ± 0.06 for HL (Table 4). The moderate heritability of these traits indicated that selection for higher body weight and length and larger skin size might be possible through genetic selection. Thirstrup et al. [6] estimated the heritability of 0.45 for both male and female skin size, which was similar to the heritability (±SE) obtained for skin size in our study (0.41 ± 0.06). However, Lagerkvist et al. [25] reported higher heritability for skin size (0.57). Lagerkvist et al. [25] only used the records collected from one sex (male) that have been under selection for five generations to estimate the genetic parameters. Moreover, the selection for male body weight might influence the genetic variation of this trait due to allele frequency changes. Therefore, the statistical models and population characteristics might be the potential reasons leading to these discrepancies.

Heritabilities (±SE) estimated for DNAP, DQU, LNAP and LQU were equal to 0.23 ± 0.10, 0.12 ± 0.04, 0.44 ± 0.07 and 0.28 ± 0.06, respectively (Table 4). Low to moderate heritabilities for these traits revealed that there is a potential to improve these fur characters using genetic and genomic selection. Among sliver blue mink in China, the estimated heritability for LNAP is 0.52 [18], which is higher than our result (0.44 ± 0.07). In blue fox (*Alopex lagopus*), the heritability of LNAP is estimated at 0.19 [26], which is lower than our estimated heritability for this trait. Berg [27] estimated that the heritability for mink pelt guard hair length ranged from 0.22 to 0.34, which is comparable with the result of our study (0.23 ± 0.10). The heritability for DNAP in blue fox is estimated to be 0.36 [28], which is higher than our estimate (0.23 ± 0.10). Difference in genetic background and environmental factors between the two species might be the potential reasons for difference in estimated heritability. The heritability reported for LQU ranges from 0.19 to 0.35 in previous studies of American mink [6,14,15,25,29], which is comparable with our result (0.28 ± 0.06). Thirstrup et al. [6] estimated a heritability of 0.30 for DQU, which is higher than our estimate of 0.12 ± 0.04 for this trait. In our study, we had four categories, while Thirstrup et al. [6] defined 12 categories for this trait. Additionally, DQU evaluation is being subjectively performed by fur evaluators, and there is no universal definition for DQU available in the literature. Therefore, it is possible that slightly different criteria were used by Thirstrup et al. [6] for the definition of DQU compared with those in our study.

### 3.4. Genetic and Phenotypic Correlations between Dried Pelt, and Body Weight and Length Traits

Phenotypic and genetic correlations between body weight, body length and pelt traits are shown in Table 4. High genetic correlations were estimated between DPS and Nov_BW (0.89 ± 0.10), Nov_BL (0.81 ± 0.07), HW (0.85 ± 0.05) and HL (0.85 ± 0.06). These results indicated that both body weight and length traits could be used as reliable measurements to predict the dried pelt size for the market. Considering the strong genetic correlations of Nov_BW with HW (0.99 ± 0.01) and Nov_BL with HL (0.86 ± 0.05), body weight and body length measured in November of the first year of life could be used as good indicators for indirect selection of the final skin size of mink. This can be particularly beneficial for mink farmers because the selection of breeders for the next breeding season is usually performed in November. This result suggested that body weight and length in November of the first year of life would be reliable indicators for market pelt size, which is a determinant of the final price of skin and subsequently the profitability of mink producers.

All phenotypic correlations between DQU and body weights and lengths in November and harvest time were not significant (*p* < 0.05). However, DQU had positive genetic correlations with Nov_BL (0.55 ± 0.24) and HL (0.46 ± 0.20), suggesting that body length traits might be good indicator traits to improve both dried pelt size and overall quality. On the other hand, the genetic correlations of DQU with Nov_BW (0.25 ± 0.25) and HW (0.06 ± 0.20) were not significant (*p* < 0.05). However, Thirstrup et al. [6] reported significant negative genetic correlations between HW and DQU (ranging from −0.38 to −0.52). This discrepancy might be due to the differences in the definition of traits and scores applied to measure this trait in these studies. In our study, DQU had four categories, while Thirstrup et al. [6] considered 12 different categories for DQU that might be based on different criteria compared with the categories applied in the current study.

No significant (*p* > 0.05) phenotypic or genetic correlations were estimated between DNAP and body weight and length traits, which ranged from 0.29 **±** 0.19 to 0.48 ± 0.32 for body weight and from −0.01 ± 0.30 to −0.02 ± 0.19 for body length traits. These results showed that independent genes might be involved in controlling pelt quality, and body weight and length traits suggest that selection for larger body weight and length would not have a negative impact on dried pelt nap size.

### 3.5. Genetic and Phenotypic Correlation between Live Grading and Body Weight and Length Traits

Phenotypic and genetic correlations between live grading and body weight and length traits are shown in Table 4. Nov_BW had a low positive phenotypic correlation with LNAP (0.11 ± 0.04) and LQU (0.10 ± 0.04). However, genetic correlations of LNAP and LQU with all body weight and length traits were not significantly different from zero (*p* > 0.05). These results suggested that selection for larger body size in November of the first year of life would not negatively affect live grading traits in mink. Similar to our results, Thirstrup et al. [6] reported nonsignificant genetic correlations between body weight and LQU (−0.13 to 0.18) and between body weight and LNAP (−0.01 to 0.07).

### 3.6. Genetics and Phenotypic Correlations between Dried Pelt and Live Fur Grading Traits

A strong positive genetic correlation (0.82 ± 0.22) was observed between LNAP and DNAP (Table 4). This suggested that selection for shorter DNAP based on the corresponding live grading trait could be effective in breeding programs. To our knowledge, this is the first report on the estimation of genetic correlation between DNAP and LNAP in mink; therefore, no previous study was available for comparison. There was a moderate genetic correlation between LNAP and LQU (0.45 ± 0.12), which was lower than the value (0.86) estimated previously in the literature [6]. Differences in scoring scales, statistical models and population structure might be responsible for this difference. LNAP and LQU were scored in five categories in the Thirstrup et al. [6] study; however, LNAP had five and LQU had three categories in our study. Moreover, the fixed effect of color type was significant for nap size traits in the present study. However, all mink used in Thirstrup et al. [6] were from the same color type (i.e., standard dark brown), so no fixed effect of the color type needed to be specified in their statistical model for estimation of genetic correlations. The genetic correlation of the LQU and DQU (0.08 ± 0.45) was not significant in the present study. Lagerkvist et al. [11] reported a nonsignificant genetic correlation of 0.15 ± 0.16 between underfur density on live mink and dried pelt, which was in accordance with our result. In our study, the density of underfur was the main criteria for classification of pelts in different quality categories. Kaszowski et al. [30] found the average underfur density for live mink to be approximately 34 percent lower than the underfur density in dried pelts, which might be due to shrinkage of pelts during the drying process leading to a change in the density of hairs relative to the pelt surface on the skins.

Indirect selection for pelt traits based on live grading is in demand by mink breeders since they can be measured on selection candidates. In the present study, high positive genetic correlation was estimated between LNAP and its corresponding dried pelt trait (0.82 ± 0.22) and between body weight and length traits with DPS ranging from 0.81 ± 0.07 to 0.89 ± 0.10. However, the genetic and phenotypic correlations between LQU and DQU were poor and nonsignificant. Therefore, selection based solely on live grading may not lead to the maximum improvement for dried pelt characters in mink. Peura et al. [26] suggested using a multi-trait selection approach to estimate the breeding values for candidate selection by combining information from live grading and pelt traits recorded on the relatives of selection candidates for improving pelt traits in blue foxes. However, multi-trait selection may not be an effective method for pelt traits in our mink population. The first reason is the nonsignificant genetic correlation between LQU and DQU (0.08 ± 0.45) estimated in our study. Moreover, when information from relatives is used for the estimation of breeding value, the selection accuracy will be considerably lower than the accuracy of the breeding value estimated based on the animals’ own performance, which would lead to a lower selection response and genetic improvement [31]. An alternative method can be selecting animals based on their genomic breeding values directly estimated from genotype information obtained for pelt traits. Genomic selection has proved to be particularly beneficial to select for traits that are measured post-mortem [32,33,34]. The correlated traits evaluated in the current study such as body weight and body length with DPS, body length in November with DQU and live grading nap size with dried pelt nap size along with molecular information can be used to obtain more accurate estimates of breeding value for pelt quality traits using multi-trait genomic prediction.

Our study provides insights into the proportion of genetic and environmental sources of phenotypic variation in dried pelt, live grading and body weight and body length traits in American mink. The absence of live grading records for Millbank farm restricted the number of available records for these traits, which might impact the genetic correlations between live grading traits and pelt traits in our study. The estimated genetic parameters in this study provided the basic knowledge for designing the genetic selection programs for fur quality in Canadian mink populations.

## 4. Conclusions

Genetic selection for fur quality and skin size can increase the production efficiency and consequently the economic profits of mink farmers. The present study was the first estimation of genetic parameters for fur quality traits in Canadian mink populations. Estimated moderate heritabilities for pelt, live grading and body weight and length traits suggested that improvement of these traits is possible using genetic/genomic selection. The estimated genetic parameters showed the potential of Nov_BW and Nov_BL as good indicators of DPS without negative effects on DQU and DNAP. The presence of moderate positive genetic correlations between body length in November and harvest with DQU makes these traits suitable for select to increase DPS and DQU. In addition, live grading nap size is a reliable indicator of dried pelt nap size. The results established a foundation for a more efficient selective breeding method for the mink industry that can be incorporated into a multi-trait genetic or genomic selection program.

## Figures and Tables

**Table 1:** Significance of fixed and random effects included in the models for dried pelt, live grading and body weight and length traits in mink.

Traits ^1^	Fixed Effects					Random Effects	
	Farm	Sex	Color Type	Year	Age	Common Litter	Maternal Genetic
DPS	*	*	NS	*	NT	NT	NS
DNAP	*	NS	*	*	NT	NT	*
DQU	*	NS	NS	*	NT	NT	NS
LNAP	NT	NS	*	*	*	NT	*
LQU	NT	NS	*	*	*	NT	NS
Nov_BW	*	*	NS	*	NT	NT	*
Nov_BL	*	*	*	*	NT	NT	*
HW	*	*	NS	*	NT	NS	NS
HL	*	*	*	*	NT	*	NS

^1^ DPS = dried pelt size; DNAP = dried pelt nap size; DQU = overall quality of dried pelt; LNAP = live grading nap size; LQU = live grading overall quality of fur; Nov_BW = November body weight; Nov_BL = November body length; HW = harvest body weight; HL = harvest body length, NT: not tested; NS: not significant. * *p* < 0.05.

**Table 2:** Descriptive statistics for dried pelt, live grading and body weight and length traits in mink.

Traits ^1^	Number of Records	Mean	Standard Deviation	Range	Coefficient of Variation (%)
DPS	1195	5.71	1.70	1–9	29.82
DNAP	1125	6.17	1.40	1–8	22.69
DQU	1191	3.40	0.78	1–4	22.87
LNAP	1608	3.07	0.95	1–5	30.94
LQU	1607	2.02	0.71	1–3	35.14
Nov_BW (gr)	1734	2.18	0.68	0.92–3.86	31.19
Nov_BL (cm)	1734	39.57	4.68	31–51	11.82
HW (gr)	2162	2.24	0.75	0.79–4.1	33.48
HL (cm)	2162	45.38	5.03	35.5–59	11.08

^1^ DPS = dried pelt size; DNAP = dried pelt nap size; DQU = overall quality of dried pelt; LNAP = live grading nap size; LQU = live grading overall quality of fur; Nov_BW = November body weight; Nov_BL = November body length; HW = harvest body weight; HL = harvest body length.

**Table 3:** Variance components and heritabilities (±SE) estimated using univariate models for dried pelt traits, live grading and body weight and length traits in mink.

	Variance Components ^2^				Genetic Parameters ^3^		
Traits ^1^	$\sigma_{a}^{2}\pm SE$	$\sigma_{c}^{2}\pm SE$	$\sigma_{m}^{2}\pm SE$	$\sigma_{e}^{2}\pm SE$	$h_{a}^{2}$	$c^{2}$	$h_{m}^{2}$
DPS	0.24 ± 0.04	NT	NS	0.35 ± 0.03	0.41 ± 0.06	NA	NA
DNAP	0.21 ± 0.10	NT	0.14 ± 0.05	0.59 ± 0.06	0.22 ± 0.10	NA	0.15 ± 0.06
DQU	0.07 ± 0.03	NT	NS	0.51 ± 0.03	0.12 ± 0.04	NA	NA
LNAP	0.26 ± 0.05	NT	0.04 ± 0.02	0.32 ± 0.03	0.42 ± 0.06	NA	0.07 ± 0.03
LQU	0.11 ± 0.02	NT	NS	0.37 ± 0.02	0.23 ± 0.5	NA	NA
Nov_BW	0.016 ± 0.73 × 10^−2^	NT	0.015 ± 0.37 × 10^−2^	0.047 ± 0.42 × 10^−2^	0.21 ± 0.09	NA	0.19 ± 0.04
Nov_BL	0.92 ± 0.33	NT	0.51 ± 0.16	2.02 ± 0.19	0.27 ± 0.09	NA	0.15 ± 0.04
HW	0.04 ± 0.57 × 10^−2^	NS	NS	0.05 ± 0.41 × 10^−2^	0.44 ± 0.09	NA	NA
HL	1.32 ± 0.34	0.45 ± 0.16	NS	3.29 ± 0.22	0.26 ± 0.06	0.09 ± 0.03	NA

^1^ DPS = dried pelt size; DNAP = dried pelt nap size; DQU = overall quality of dried pelt; LNAP = live grading nap size; LQU = live grading overall quality of fur; Nov_BW = November body weight; Nov_BL = November body length; HW = harvest body weight; HL = harvest body length. ^2^$\;\sigma_{a}^{2}$ = additive genetic variance; $\sigma_{m}^{2}$ = maternal genetic variance; $\sigma_{c}^{2}$ = common litter variance; $\sigma_{e}^{2}$ = residual variance. ^3^$\;h_{a}^{2}$ = heritability from univariate models; $c^{2}$ = proportion of phenotypic variance explained by common litter effects; $h_{m}^{2}$ = proportion of phenotypic variance explained by maternal genetic effects. NS: not significant (*p* > 0.05); NA: not applicable; NT: not tested.

**Table 4:** Estimated heritabilities (±SE) (diagonal), genetic correlations (above diagonal) and phenotypic correlations (below diagonal) for dried pelt, live grading and body weight and length traits in mink.

**Traits** **^1^**	DPS	DNAP	DQU	LNAP	LQU	Nov_BW	Nov_BL	HW	HL
DPS	**0.41** **±** **0.06**	0.26 ± 0.20	0.26 ± 0.21	0.04 ± 0.20	−0.14 ± 0.32	**0.89 ± 0.10**	**0.81 ± 0.07**	**0.85 ± 0.05**	**0.85 ± 0.06**
DNAP	0.01 ± 0.03	**0.23 ± 0.10**	0.13 ± 0.25	**0.82 ± 0.22**	0.42 ± 0.34	0.48 ± 0.32	−0.01 ± 0.30	0.29 ± 0.19	−0.02 ± 0.19
DQU	0.01 ± 0.03	**0.14 ± 0.03**	**0.12 ± 0.04**	0.15 ± 0.30	0.08 ± 0.45	0.25 ± 0.25	**0.55 ± 0.24**	0.06 ± 0.20	**0.46 ± 0.20**
LNAP	0.01 ± 0.07	**0.45 ± 0.06**	0.06 ± 0.06	**0.44 ± 0.07**	**0.45 ± 0.12**	0.20 ± 0.19	0.12 ± 0.25	0.09 ± 0.21	0.15 ± 0.26
LQU	−0.07 ± 0.07	**0.29 ± 0.06**	0.01 ± 0.06	**0.23 ± 0.02**	**0.28 ± 0.06**	−0.04 ± 0.20	−0.15 ± 0.22	−0.36 ± 0.21	−0.28 ± 0.25
Nov_BW	**0.64 ± 0.01**	0.06 ± 0.04	−0.01 ± 0.03	**0.11 ± 0.04**	**0.10 ± 0.04**	**0.29 ± 0.10**	**0.79 ± 0.12**	**0.99 ± 0.01**	**0.53 ± 0.02**
Nov_BL	**0.50 ± 0.03**	−0.06 ± 0.04	0.03 ± 0.03	−0.08 ± 0.04	−0.04 ± 0.04	**0.55 ± 0.02**	**0.28 ± 0.09**	**0.90 ± 0.05**	**0.86 ± 0.05**
HW	**0.69** **±** **0.01**	0.07 ± 0.03	−0.02 ± 0.03	0.04 ± 0.06	0.01 ± 0.06	**0.83 ± 0.01**	**0.52 ± 0.02**	**0.41 ± 0.07**	**0.83 ± 0.05**
HL	**0.49 ± 0.02**	0.005 ± 0.3	0.01 ± 0.03	0.04 ± 0.06	−0.02 ± 0.05	**0.53 ± 0.02**	**0.51 ± 0.02**	**0.53 ± 0.01**	**0.31 ± 0.06**

^1^ DPS = dried pelt size; DNAP = dried pelt nap size; DQU = overall quality of dried pelt; LNAP = live grading nap size; LQU = live grading overall quality of fur; Nov_BW = November body weight; Nov_BL = November body length; HW = harvest body weight; HL = harvest body length. Significant heritabilities and correlations are highlighted in bold (*p* < 0.05).

## Data Availability

Data may be available upon request by contacting the corresponding author.
